# How to tackle chemical communication? Relative proportions versus semiquantitative determination of compounds in lizard chemical secretions

**DOI:** 10.1002/ece3.3825

**Published:** 2018-01-17

**Authors:** Roberto García‐Roa, Jorge Sáiz, Belén Gómara, Pilar López, José Martín

**Affiliations:** ^1^ Departamento de Ecología Evolutiva Museo Nacional de Ciencias Naturales Spanish Research Council (MNCN‐CSIC) Madrid Spain; ^2^ Department of Instrumental Analysis and Environmental Chemistry Spanish Research Council (IQOG‐ CSIC) Madrid Spain

**Keywords:** chemoreception, chromatography, communication, mass spectrometry, reptiles, semiochemicals

## Abstract

Knowledge about chemical communication in some vertebrates is still relatively limited. Squamates are a glaring example of this, even when recent evidences indicate that scents are involved in social and sexual interactions. In lizards, where our understanding of chemical communication has considerably progressed in the last few years, many questions about chemical interactions remain unanswered. A potential reason for this is the inherent complexity and technical limitations that some methodologies embody when analyzing the compounds used to convey information. We provide here a straightforward procedure to analyze lizard chemical secretions based on gas chromatography coupled to mass spectrometry that uses an internal standard for the semiquantification of compounds. We compare the results of this method with those obtained by the traditional procedure of calculating relative proportions of compounds. For such purpose, we designed two experiments to investigate if these procedures allowed revealing changes in chemical secretions 1) when lizards received previously a vitamin dietary supplementation or 2) when the chemical secretions were exposed to high temperatures. Our results show that the procedure based on relative proportions is useful to describe the overall chemical profile, or changes in it, at population or species levels. On the other hand, the use of the procedure based on semiquantitative determination can be applied when the target of study is the variation in one or more particular compounds of the sample, as it has proved more accurate detecting quantitative variations in the secretions. This method would reveal new aspects produced by, for example, the effects of different physiological and climatic factors that the traditional method does not show.

## 1 INTRODUCTION

Multiple modes of animal communication have been investigated to improve the knowledge of animal ecology and evolution (Bradbury & Vehrencamp, 2011). However, what we currently know about communication in vertebrates derives mostly from studies on acoustic and visual interactions (Espmark, Amundsen, & Rosenqvist, 2000; Hauser & Konishi, 2003; Osorio & Vorobyev, 2008). In fact, other alternative modes of conveying information, such as through chemical cues and signals, have been dramatically overlooked in vertebrates, whereas in arthropods, for instance, the study of the molecules used in chemical interactions has provided crucial keys to understand the underlying process and mechanism to their social and sexual behaviors (Wyatt, 2014).

Squamates often own well‐developed olfactory and vomeronasal organs that function in chemoreception (Halpern, 1992) to detect and discriminate prey, predators, and conspecifics (Houck, 2009; Mason & Parker, 2010). On the latter point, chemical signals are thought to have leading roles in mate choice and intrasexual competition processes (Mason, 1992). Lizards, in particular, can have a series of epidermal holocrine glands that protrude a waxy secretion employed for scent marking (Mayerl, Baeckens, & Van Damme, 2015). These glands are located in the femoral, or in the precloacal region or forming continuous rows between both areas of the body (Baeckens, Edwards, Huyghe, & Van Damme, 2015; García‐Roa, Jara, et al., 2017; Valdecantos, Martínez, & Labra, 2014). The produced chemical secretion contains useful information for lizard social behaviors (Martín & López, 2015; Mason & Parker, 2010; Olsson et al., 2003). In these secretions, lipids are the main compounds together with proteins (García‐Roa, Megía‐Palma, et al., 2017; Ibáñez et al., 2017; Khannoon, 2016; Mangiacotti et al., 2016). Some studies have revealed that quantitative variations in the lipophilic fraction of secretions may produce different responses on receiver individuals (Kopena, Martín, López, & Herczeg, 2011; Martín & López, 2013). Given that some behaviors of male–male competition or female mate choice could be highly influenced by changes in certain compounds (e.g., tocopherols, cholesterol) (reviewed in Martín & López, 2015), an accurate detection of these quantitative variations will be crucial to gain a better grasp of how chemical signaling operates.

The procedures used to investigate chemical ecology in lizards have differed depending on the research group and the purpose of the study. Many works have been based on behavioral approaches alone, for example, studying differences in tongue flick rates or preferences for scent‐marked areas (Baird, McGee, & York, 2015; Cooper & Steele, 1997). For the past two decades, studies using analytical techniques have been often employed. For instance, to investigate the composition of chemical secretions, gas chromatography coupled to mass spectrometry (GC‐MS) is currently the most common analytical technique (Flachsbarth, Fritzsche, Weldon, & Schulz, 2009; Heathcote, Bell, d'Ettorre, While, & Uller, 2014; Khannoon et al., 2011; Louw, Burger, Le Roux, & Van Wyk, 2007, 2011). However, procedures may vary in terms of the sample treatment and analysis. The secretions are frequently subjected to a solid–liquid extraction step by adding solvents to the vial containing the secretion (Gabirot, Lopez, & Martín, 2012; Kopena, López, & Martín, 2009). The extracted compounds can be subsequently derivatized by adding reagents to the sample for replacing the active points in the molecules by nonpolar groups, in order to improve the detectability of some types of compounds, such as fatty acids (Khannoon et al., 2011). After separation of compounds in the GC, the mass spectra resulting from the MS analysis can be identified using mass spectra libraries (Stein, 2012) and by comparing the retention times and mass spectra with commercial standards available from chemicals supply companies (Ibáñez et al., 2017; Louw et al., 2007). In this process, analyses made on GC‐MS usually operate the MS in scan mode to identify the highest number of compounds in the sample as possible (García‐Roa, Carreira, López, & Martín, 2016; Khannoon, 2012; Martín et al., 2016; Runemark, Gabirot, & Svensson, 2011). Final results provide the relative proportion of each peak (i.e., compound) expressed as a percentage of the peak area considered in the total ion chromatogram (TIC) (Escobar, Escobar, Labra, & Niemeyer, 2003; Heathcote et al., 2014; Khannoon, 2016; Runemark et al., 2011), which entails certain limitations. As the percentage value is based on the TIC, an increase in the area of a given compound will result in the decrease on the relative areas of the remaining compounds. This nonindependence of the compounds leads to the problem of the fluctuation in the proportions of compounds, even when their abundance does not actually change. As an example of this, four compounds are in similar proportions in a sample (25%, 25%, 25%, 25%—the sum of the proportions of the chemicals is constant and always 100%). An external factor affects the expression of the first compound, increasing its concentration. As a result, the other three compounds will decrease their relative proportions (40%, 20%, 20%, 20%), even if the external factor does not affect their actual abundances. Thus, an erroneous interpretation of these results would conclude that the external factor negatively affected the expression of the last three compounds too, while the truth is that only the first compound was affected. In this context, the use of relative proportions might make difficult to statistically identify quantitative variations among different secretions.

The aim of this study was to test an alternative procedure to investigate lizard chemical secretions, based on the semiquantification of their chemical compounds (hereafter termed as “SQ procedure”). This method allows the semiquantification of compounds by conferring quantitative independence among them, avoiding the constant‐sum problem (the sum of the relative proportions of compounds must be always 100). To test the reliability of this procedure, we designed two pilot experiments, identifying the quantitative variation, produced by the effects of physiological (dietary supplementation) and climatic (temperature) factors, in lizard chemical secretions. We compared the results obtained analyzing the relative abundances from the SQ procedure, with those obtained from the calculation of the relative proportions with respect to the total ion current (TIC), which is the method usually employed to analyze chemical secretions to date (hereafter termed as “TIC procedure”).

## 2 MATERIALS AND METHODS

We analyzed the chemical secretions of two lizard species, *Iberolacerta cyreni* and *Psammodromus algirus*. For both experiments, all lizards were captured by noosing and released healthy at the end of the experiments at their capture sites.

The first experiment involved male Carpetane rock lizards (*I. cyreni*) captured at “Alto del Telégrafo” (40°47′N, 04°00′W, Sierra de Guadarrama, Madrid, Spain) in the spring of 2015. They were divided into control males (*n* = 19) and treated males (*n* = 19). The treated males were orally supplemented using plastic syringes with 5 μl of vitamin E (i.e., α‐tocopherol; from Sigma‐Aldrich Chemicals Co.), whose composition is 97% of synthetic vitamin E (approx. 1014 IU ml21) and 3% soybean oil (with approx. 0.32 IU ml21 of natural vitamin E, i.e., D‐α‐tocopherol). This dietary supplementation was conducted every 2 days during 3 weeks in June 2015. A similar procedure was followed during the same period of time for control lizards, but supplementing them with 5 μl of soybean oil alone (García‐Roa, Sáiz, Gómara, López, & Martín, 2017; Kopena et al., 2011). With this experiment, we aimed to assess whether the SQ procedure was able to detect quantitative changes in the relative abundances of certain compounds found in secretions produced by the effect of an external factor (dietary supplementation) exerted on individuals. The supplementation in the diet of some vitamins may lead to physiological changes that can later be partially expressed in qualitative and qualitative variations in the composition of chemical secretions (see examples in Martín & López, 2015). Given the high number of compounds found in femoral secretions of *I. cyreni* (López & Martín 2005), we selected, as an example, a set of them representing the different major classes of compounds found as follows: aldehydes (tetradecanal), steroids (cholesta‐3, 5‐diene, cholesterol, campesterol, lanost‐8‐en‐3‐ol, and β‐sitosterol), and tocopherols (α‐tocopherol). Moreover, these compounds encompass different relative abundances, ranging from low (e.g., lanost‐8‐en‐3‐ol) to high (cholesterol) presence, ensuring that the procedure can be used to study a wide array of compounds.

In the second experiment, we captured 18 males of Algerian Psammodromus lizards (*P. algirus*) at “La Golondrina” oak forest near Navacerrada village (40°43′N, 04°01′W; Sierra de Guadarrama, Madrid, Spain). Their chemical secretions were extracted and divided into two halves. The first one was exposed to 28°C in an incubator for 3 h prior its conservation at −20°C in a freezer. The other half of the secretion was kept directly in the freezer after the extraction as a control. As chemical secretions that suffer high environmental temperatures after being deposited on a substrate may have a lower detectability by lizards (Martín & López, 2013), we aimed to test whether the SQ procedure allowed detecting quantitative changes in certain selected compounds (i.e., hexadecanoic acid, 9,12‐octadecadienoic acid, octadecanoic acid, squalene, cholesta‐3,5‐diene, cholesterol, ergosterol, campesterol, stigmasterol, and β‐sitosterol). In this case, the compounds selected were chosen because they represented a variety of the different chemical major classes of lipids found in secretions of *P. algirus* (Martín & López, 2006). However, as in the previous experiment, other compounds could be considered in the analysis.

### 2.1 Secretion collection

**Figure 1:**
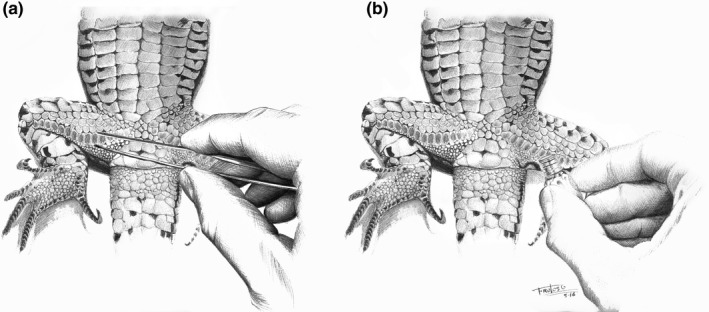
Extraction of chemical secretions of lizards. (a) Secretion collection after gently pressing with forceps around the lizard's femoral pores. (b) Direct collection of secretions by pressing glass vials against the lizard's femoral pores. Both methods may be also used for the extraction of precloacal pores secretions

All the materials used for the secretion extraction were cleaned with *n*‐hexane (99%, J.T. Baker; Deventer, The Netherlands) in order to remove organic compounds. Then, we extracted the secretions from live animals using sterile forceps pressing gently around the pores until collecting the sample in a vial (Figure 1a). However, a direct extraction pressing the glass vial over the pores is also possible (Figure 1b). Blank control vials were also prepared in order to compare with those containing samples and to exclude potential events of contamination from handling procedures during sampling and analytical processes. Extracted secretions were directly introduced into total recovery glass vials (1.1 ml, Análisis Vínicos S.L., Tomelloso, Spain, ref. V2275) closed with Teflon‐lined stoppers. Vials were stored at ‐20°C to avoid sample degradation because of volatility of some chemicals and potential bacterial activity that may change the composition of secretions (Canuel & Martens, 1996).

### 2.2 Sample preparation

**Figure 2:**
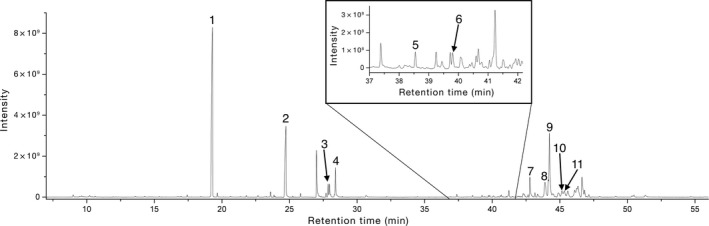
Chromatogram showing the separation of compounds present in secretions of a Psammodromus algirus male lizard (Fam. Lacertidae). 1—*n*‐heptadecane (internal standard); 2—*n*‐hexadecanoic acid; 3—9,12‐octadecadienoic acid; 4—octadecanoic acid; 5—squalene; 6—cholesta‐3,5‐diene; 7—cholesterol; 8—campesterol; 9—ergosterol; 10—stigmasterol; 11—β‐sitosterol

In the analytical laboratory, samples were removed from the vials using tweezers and dissecting needles and were weighed using a XP2U ultra‐microbalance (Mettler Toledo; Columbus, OH. Accurate readability: 0.0001 mg). Disposable microaluminum foil dishes with rims were used for weighing. During the weighing process, the temperature in the room was kept at 20°C. All the laboratory supplies (tweezers, dissecting needles, etc.) were cleaned with *n*‐hexane (99%, J.T. Baker; Deventer, the Netherlands) before and after each weighing measurement. *n*‐Hexane was also used as solvent for solid–liquid extraction. In addition, *n*‐heptadecane (99%, Sigma‐Aldrich; St Louis, MO) was added to the samples as an internal standard (IS) (Figure 2). For this, a solution of 50 ppm of *n*‐heptadecane dissolved in *n*‐hexane was prepared. Then, 1 μl of this solution was added to the sample per each 20 μg of femoral secretion. The mixture was vortex‐mixed for 2 min and left it in the fridge (4°C) for the precipitation of solid particles. Five minutes later, the liquid phase was collected with a micropipette and transferred to a total recovery glass vial suitable for GC analysis, which were closed with Teflon‐lined stoppers, and this was called the sample. Finally, the samples were kept at −20°C until their analysis. This methodology can be constrained by the minimum amount of sample required for the analyses; as for samples of less than 0.3 mg, the volume of solvent needed was too low for preparation and injection in the chromatographic system. However, the secretions analyzed in this work weighed an average of 1.167 mg, which far exceed the minimum required and can be representative of the amount available in many lizard species.

### 2.3 Analysis of samples

Analyses were performed with a TRACE GC Ultra gas chromatograph (GC) coupled to a mass spectrometer (MS) equipped with a triple quadrupole analyzer (TSQ Quantum XLS, Thermo Fisher Scientific Inc.; Bremen, Germany) operated in electron impact ionization mode (EI, −70 eV of electron energy). The mass spectrometer was operated in scan detection mode. The current of the filament was 150 μA. A volume of 2 μl of each sample was injected in the analytical system. Injections were performed in a programmed temperature vaporization (PTV) injector in splitless mode. The split flow was 10 ml/min, and the splitless time was 2 min. The injector was kept at 250°C during the injection and transfer phases with a constant septum purge. We included a cleaning phase of the injector after the transfer phase consisting in an increase in temperature of the injector at 14.5°C/s up to 350°C. Then, the temperature was held at 350°C for 5 min. The flow was also increased up to 50 ml/min in the cleaning phase. A capillary column HP‐5MS (30 m × 0.25 mm i.d., 0.25 μm film thickness) purchased from Agilent Technologies (Palo Alto, CA, USA) was used for the separation. An initial temperature of 100°C for 3 min was programmed in the GC oven, and it was increased at 5°C/min to 300°C. The final temperature was held for 15 min. Helium was used as the carrier gas at a constant flow rate of 0.8 ml/min. The temperature of the transfer line and the MS source was set at 300°C and 240°C, respectively. Data recording was started 7 min after the separation began, as solvent delay and in order to increase the filament life. We used Xcalibur™ 2.1.0.1140 software (Thermo Fischer Scientific Inc., San Jose, CA, USA) to record and process the acquired data. Finally, we identified the chemical components by comparing their mass spectra with those of compounds provided by the NIST/EPA/NIH (NIST 02) computerized mass spectral library. Commercial standards were compared with spectra and retention times of our compounds to confirm the identification. The repeatability and intermediate precision of the analytical method were studied for the IS, as it is the unique compound present in all the samples at the same concentration. The repeatability was studied for five consecutive injections, being the relative standard deviation (RSD) 0.04% for the retention times and 1.7% (RSD) for peak areas. The intermediate precision was studied in four nonconsecutive days over 8 days and was 0.2% (RSD) for the retention times and 6.7% (RSD) for the peak areas.

### 2.4 Statistical procedures

We first calculated the relative proportion of each compound in femoral secretions as the percent of the total ion current (TIC) area, excluding the IS area. To correct the problem of nonindependence of proportions, we transformed areas following Aitchison's formula: [*Z*_ij_ = ln(*Y*_ij_/g(*Y*_j_)], where *Z*_ij_ is the standardized peak area i for individual j, *Y*_ij_ is the peak area i for individual j, and g(*Y*_j_) is the geometric mean of all peaks for individual j (Aitchison, 1986) (for similar procedures see for example (López, Amo, & Martín, 2006).

For the semiquantitative analyses, the areas of all the analytes (*A*_*anal*_) were expressed as a percentage (*RA,* relative abundance) of the IS peak area (*A*_*stand*_). That is, *RA* = *(A*_*anal*_ */A*_*stand*_ *) × 100*. We log‐transformed the data resulting from semiquantification of each compound to ensure normality (Shapiro–Wilk's test). Tests of homogeneity of variances (Levene's test) showed that heterogeneity of variances was not significant in all cases.

We conducted general linear models (GLM) to study the effect of the dietary supplementation of vitamin E in *I. cyreni* on the relative proportion and relative abundance of each compound. To assess potential changes in the relative proportion and relative abundance of the selected compounds due to the effect of temperature in the deposited chemical secretions of *P. algirus,* we used repeated measures general linear models (GLM) with the temperature treatment (control vs. increased) applied to different portions of the secretion of the same individual lizard as a within factor. All the statistical analyses were conducted with STATISTICA version 8.0 and SPSS 20.0.0.

## 3 RESULTS

### 3.1 Vitamin E supplementation

**Figure 3:**
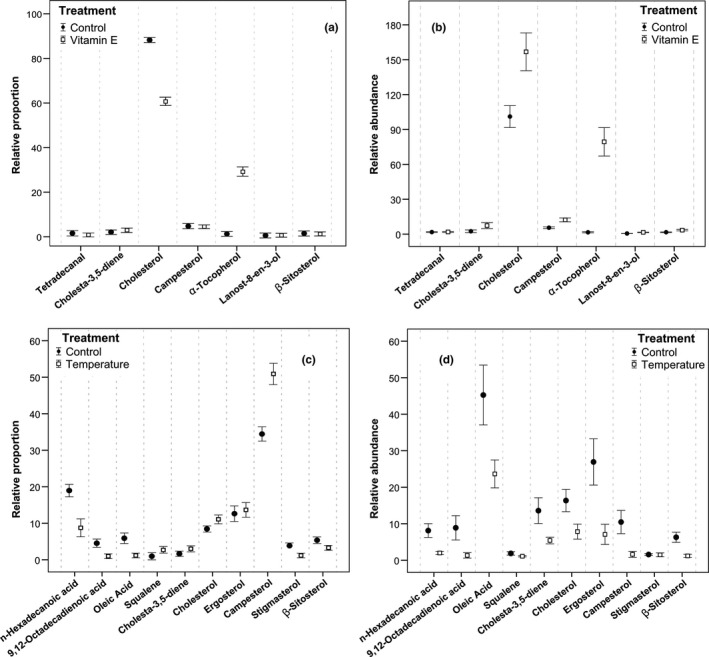
Effects of two different treatments on the chemical composition of lizard femoral secretions. Intensity (mean ± SE) of seven particular compounds in secretions of control and vitamin E diet supplemented males of *Iberolacerta cyreni* obtained from (a) the calculation of the relative proportions with respect to the total ion current (TIC procedure) and (b) from the semiquantification of compounds using an internal standard (SQ procedure). Intensity (mean ± SE) of ten particular compounds in control and temperature‐treated secretions of *Psammodromus algirus* male lizards resulting from the analysis with (c) the TIC procedure and (d) the SQ procedure

Considering the relative proportion of compounds respect to the total TIC area, secretions of supplemented males showed significant overall differences compared to those of control males (GLM comparing relative proportions of seven compounds; Wilks's λ = 0.34, *F*_6,31_ = 9.87, *p *<* *.001) (Figure 3a). However, the effect of the vitamin E dietary supplementation differed among compounds. Thus, relative proportions of α‐tocopherol increased highly and significantly after the supplementation (*F*_1,36_ = 38.43, *p *<* *.001) and the relative proportions of lanost‐8‐en‐3‐ol also showed a small but significant increase (*F*_1,36_ = 15.79, *p *<* *.001). In contrast, relative proportions of cholesterol decreased highly and significantly after the supplementation (*F*_1,36_ = 28.75, *p *<* *.001) and proportions of campesterol (*F*_1,36_ = 16.83, *p *<* *.001) and β‐sitosterol (*F*_1,36_ = 21.24, *p *<* *.001) also decreased significantly, although in lower proportions. However, the diet supplementation did not lead to significantly different changes in relative proportions of tetradecanal (*F*_1,36_ = 2.82, *p* = .10) and cholesta‐3,5‐diene (*F*_1,36_ = 0.04, *p *=* *.84).

Considering the semiquantitative determination of compounds, there were also overall significant differences between secretions of supplemented males and those of control males (GLM comparing relative abundances of seven compounds; Wilks's λ = 0.10, *F*_7,30_ = 36.50, *p *<* *.001) (Figure 3b). The effect of the vitamin E dietary supplementation differed among compounds, but changes occurred in the same direction. Thus, α‐tocopherol (*F*_1,36_ = 236.45, *p *<* *.001), campesterol (*F*_1,36_ = 18.27, *p *<* *.001), and lanost‐8‐en‐3‐ol (*F*_1,36_ = 22.79, *p *<* *.001) were highly influenced by the supplementation, increasing their relative abundances in samples of supplemented males compared to control males. Also, β‐sitosterol (*F*_1,36_ = 10.20, *p* = .002), cholesterol (*F*_1,36_ = 11.07, *p* = .002), and cholesta‐3,5‐diene (*F*_1,36_ = 4.48, *p *=* *.04) had smaller but significant higher relative abundances in the secretions of supplemented males than in control males. However, only in the case of tetradecanal, the diet supplementation did not lead to significantly different changes in its relative abundance (*F*_1,36_ = 0.02, *p *=* *.87).

### 3.2 Temperature effects on the secretions

Considering the relative proportions of compounds, the effects of the temperature treatment differed among compounds (Figure 3c). Thus, relative proportions of 9,12‐octadecadienoic acid (repeated measures GLM. *F*_1,16_ = 12.77, *p *=* *.0025), octadecanoic acid (*F*_1,16_ = 4.72, *p *=* *.04), and β‐sitosterol (*F*_1,16_ = 23.56, *p *<* *.001) decreased significantly after the temperature treatment. In contrast, relative proportions of cholesta‐3,5‐diene (*F*_1,16_ = 11.87, *p *=* *.003), cholesterol (*F*_1,16_ = 12.91, *p *=* *.002), ergosterol (*F*_1,16_ = 17.62, *p *<* *.001), and campesterol (*F*_1,16_ = 19.27, *p *<* *.001) increased significantly after the temperature treatment. However, the treatment did not significantly affect to the relative proportions of *n*‐hexadecanoic acid (*F*_1,16_ = 1.82, *p *=* *.20), squalene (*F*_1,16_ = 2.99, *p *=* *.10), or stigmasterol (*F*_1,16_ = 2.89, *p *=* *.11).

Considering the semiquantitative determination of compounds, the effects of the temperature treatment were similar for most compounds (Figure 3d). Thus, relative abundances of *n*‐hexadecanoic acid (repeated measures GLM; *F*_1,16_ = 23.14, *p *<* *.001), 9,12‐octadecadienoic acid (*F*_1,16_ = 19.23, *p *<* *.001), octadecanoic acid (*F*_1,16_ = 9.24, *p* = .007), cholesta‐3,5‐diene (*F*_1,16_ = 9.89, *p *=* *.006), cholesterol (*F*_1,16_ = 7.34, *p *=* *.01), ergosterol (*F*_1,16_ = 12.65, *p *=* *.002), campesterol (*F*_1,16_ = 9.74, *p *=* *.009), stigmasterol (*F*_1,16_ = 29.38, *p *=* *.009), and β‐sitosterol (*F*_1,16_ = 23.56, *p *<* *.001) decreased after the temperature treatment. However, the temperature treatment did not significantly affect to the relative abundance of squalene (*F*_1,16_ = 0.36, *p *=* *.149).

## 4 DISCUSSION

The implementation of methodologies to analyze lizard chemical secretions as accurately as possible is crucial to increase our understanding of ecology and evolution of the communication in vertebrates. The better the implemented methodology, the more the precision to detect changes or identify compounds in chemical secretions, which in turn provides a more realistic framework about how chemical communication functions. Both of our pilot experiments confirmed the reliability of the SQ procedure to describe the profile of chemical secretions and detect quantitative variations in their compounds.

Two different studies were designed to test the validity of the SQ procedure to analyze lizard chemical secretions and to compare the obtained results based on both the TIC procedure and the SQ procedure. With the first experiment, we confirmed the reliability of the SQ procedure to study the physiological effects produced by the dietary supplementation of vitamin E in the chemical secretions. Indeed, the vitamin E supplementation had a direct effect on the chemical profiles. However, the two methodologies showed very contrasting interpretations of these effects. We observed that, based on the TIC procedure, there was a high increase in relative proportion of vitamin E, which produced a concomitant decrease in relative proportions of other compounds (mainly cholesterol). In contrast, the SQ procedure showed that relative abundances of almost all studied compounds (all but tetradecanal) increased after the treatment. This indicates that vitamin E supplementation increased the amount of vitamin E in secretions, but also allowed or required increasing the amounts of other compounds. On the one hand, therefore, the results of both procedures reflect that supplementation modifies the chemical profiles. From an ecological point of view, it is very interesting because the “chemosensory perception”, and the subsequent behavioral response (Martín & López, 2006), by conspecific lizards could differ when receiving the chemical stimuli of both type of males (treated vs. control) (García‐Roa, Sáiz, et al., 2017; Kopena et al., 2011). However, on the other hand, when we focus on particular compounds, the statistical results and hence their interpretation of how these compounds change in both types of males are different depending on the procedure. The TIC procedure, under the statistical effect of the constant‐sum problem, showed a scenario where owing to the increase in vitamin E, the other compounds seemed to decrease. Analyzing with the SQ procedure, giving statistical independence to the compounds and avoiding the constant‐sum problem, we observed that relative abundances of all compounds but tetradecanal actually increased. This provides an opposing framework to that showed by the TIC procedure and reveals an important finding as these results highlight that the acquisition of a particular chemical may potentially alter the metabolism and produce a higher expression of other compounds through the chemical secretions, which has been suggested crucial in processes of female mate choice and territory rival assessment (Martín, Moreira, & López, 2007).

The second experiment validated the SQ procedure to detect the effect of temperature on chemical secretions. Here again, the interpretations of the results of both procedures were highly contrasting. The TIC procedure indicated that the chemical profile of temperature‐treated chemical secretions changed, by decreasing the relative proportions of fatty acids and increasing the relative proportions of steroids. In contrast, when we provided statistical independence to the compounds in the SQ procedure, we observed that all the compounds studied, except squalene, actually decreased in their relative abundances when the temperature increased. Indeed, that temperature decreases the abundance of some compounds in the chemical secretions is more coherent than an increase, which is showed by TIC procedure; the temperature would degrade the molecules, reducing their abundance in the chemical secretions. This could be the reason behind the findings of recent studies noting that, after secretions suffered a temperature treatment, the lizards perception of these secretions (measured as tongue flick rates) decreased substantially (Martín & López, 2013; Martín, Ortega, & López, 2015).

The SQ procedure described here constitutes an alternative way to study lizard chemical secretions. We believe that this method allows expanding and enhancing the ecological and evolutionary framework of lizard chemical communication, as this approach is probed to be more accurate and probably realistic to detect quantitative variations in lizard chemical secretions. Thus, while relative proportions of compounds can have roles in conspecific recognition, the use of the SQ procedure may be useful to gain insight into what is behind of dominance behaviors or mate choice processes, which have been associated by some authors to the degree of expression of specific compounds, such as α‐tocopherol or cholesterol (reviewed in Martín & López, 2015). In addition, this may facilitate the execution of new studies in the search of deeper knowledge on important topics, such as the effects of environmental (e.g., climate change) or physiological (e.g., hormones, diet) on chemical communication in lizards. In light of the results obtained here, we suggest that considerable care must be taken when choosing a methodology to analyze chemical secretions. Thus, we consider that although the TIC procedure is easier and faster to implement than the SQ procedure, it embodies some statistical limitations that must be taken into account. The TIC procedure can be useful to characterize and compare overall chemical profiles (Baeckens, García‐Roa, Martín, & Van Damme, 2017; Baeckens, Martín, García‐Roa, & van Damme, 2017b; Baeckens, Martín, García‐Roa, Pafilis, et al., 2017a; García‐Roa, Jara, López, Martín, & Pincheira‐Donoso, 2017), probably reflecting the “chemosensory perception” that a lizard may have of a secretion. Nevertheless, when the target is one or more particular compounds, it is advisable to ensure independence of these by means of including an internal standard, as has been performed in the SQ procedure (García‐Roa, Sáiz, et al., 2017). We are also aware that SQ procedure requires certain considerations. For instance, the IS must have similar chemical characteristics to the rest of the compounds, and further, it must not overlap with any of them. This can be solved, when possible, with a previous analysis of some samples based on GC‐MS operating in the MS in scan mode. This step would offer a general view of the chromatogram that would allow choosing a particular IS that meets with the requirements. On the other hand, the parameters and values in the procedure might be modified to gain effectiveness in the study of particular compounds, for example, reducing or increasing the time of signal intensity analysis in some stages of the analytical process. In fact, although the SQ procedure has been here tested using lizards as a model organism, the methodology could be also adapted to analyze chemical secretions from other vertebrates.

## CONFLICT OF INTEREST

None declared.

## AUTHOR CONTRIBUTIONS

Roberto García‐Roa conceived and designed the experiments, performed the experiments, analyzed the data, wrote the manuscript, prepared figures and/or tables, and reviewed drafts of the manuscript. Jorge Sáiz performed the experiments, analyzed the data, wrote the manuscript, and reviewed drafts of the manuscript. Belén Gomara contributed reagents/materials/analysis tools and reviewed drafts of the manuscript. Pilar López contributed reagents/materials/analysis tools and reviewed drafts of the manuscript. José Martín conceived and designed the experiments, analyzed the data, contributed reagents/materials/analysis tools, wrote the manuscript, and reviewed drafts of the manuscript.
